# A New Ex Vivo Human Skin Burn Model

**DOI:** 10.1093/jbcr/irad071

**Published:** 2023-05-18

**Authors:** Ania Labouchère, Daniel Haselbach, Murielle Michetti, Catherine Pythoud, Wassim Raffoul, Lee Ann Applegate, Nathalie Hirt-Burri, Anthony de Buys Roessingh

**Affiliations:** PlasticReconstructive, and Hand Surgery Service, Lausanne University Hospital, University of Lausanne, Lausanne, Switzerland; Regenerative Therapy Unit, Lausanne University Hospital, University of Lausanne, Lausanne, Switzerland; Lausanne Burn Center, Lausanne University Hospital, University of Lausanne, Lausanne, Switzerland; PlasticReconstructive, and Hand Surgery Service, Lausanne University Hospital, University of Lausanne, Lausanne, Switzerland; Regenerative Therapy Unit, Lausanne University Hospital, University of Lausanne, Lausanne, Switzerland; Lausanne Burn Center, Lausanne University Hospital, University of Lausanne, Lausanne, Switzerland; Regenerative Therapy Unit, Lausanne University Hospital, University of Lausanne, Lausanne, Switzerland; PlasticReconstructive, and Hand Surgery Service, Lausanne University Hospital, University of Lausanne, Lausanne, Switzerland; Lausanne Burn Center, Lausanne University Hospital, University of Lausanne, Lausanne, Switzerland; Regenerative Therapy Unit, Lausanne University Hospital, University of Lausanne, Lausanne, Switzerland; Lausanne Burn Center, Lausanne University Hospital, University of Lausanne, Lausanne, Switzerland; Center for Applied Biotechnology and Molecular Medicine, University of Zurich, Zurich, Switzerland; Regenerative Therapy Unit, Lausanne University Hospital, University of Lausanne, Lausanne, Switzerland; Lausanne Burn Center, Lausanne University Hospital, University of Lausanne, Lausanne, Switzerland; Lausanne Burn Center, Lausanne University Hospital, University of Lausanne, Lausanne, Switzerland; Children and Adolescent Surgery Service, Lausanne University Hospital, University of Lausanne, Lausanne, Switzerland

## Abstract

Currently, most burn models for preclinical testing are on animals. For obvious ethical, anatomical, and physiological reasons, these models could be replaced with optimized ex vivo systems. The creation of a burn model on human skin using a pulsed dye laser could represent a relevant model for preclinical research. Six samples of excess human abdominal skin were obtained within one hour after surgery. Burn injuries were induced on small samples of cleaned skin using a pulsed dye laser on skin samples, at varying fluences, pulse numbers and illumination duration. In total, 70 burn injuries were performed on skin ex vivo before being histologically and dermato-pathologically analyzed. Irradiated burned skin samples were classified with a specified code representing burn degrees. Then, a selection of samples was inspected after 14 and 21 days to assess their capacity to heal spontaneously and re-epithelize. We determined the parameters of a pulsed dye laser inducing first, second, and third degree burns on human skin and with fixed parameters, especially superficial and deep second degree burns. After 21 days with the ex vivo model, neo-epidermis was formed. Our results showed that this simple, rapid, user-independent process creates reproducible and uniform burns of different, predictable degrees that are close to clinical reality. Human skin ex vivo models can be an alternative to and complete animal experimentation, particularly for preclinical large screening. This model could be used to foster the testing of new treatments on standardized degrees of burn injuries and thus improve therapeutic strategies.

Burn injuries are a global public health problem and the World Health Organization estimates that 180,000 deaths per year are caused by burns every year. They are also one of the leading causes of disability and disfigurement.^1–3^ Due to the evolution of wound management and treatment since 1945, the chances of surviving burn injuries have significantly improved for patients. However, new treatments are needed to both improve patient survival rates and quality of life while reducing comorbidities, disfiguration, and psychosocial impact.

With this in mind, both in vitro and in vivo experimental models are useful. Thus, a reliable burn wound model is of critical value. However, none of the existing animal models have been found to be ideal from an anatomical, physiological, practical, and economic point-of-view. Animal experimentation tends to be replaced by models on human skin. On one hand, in vitro models are a good alternative, but they are still far from clinical reality, both from anatomical and biological perspectives.^4–6^ On the other hand, in vivo models on human skin^7–9^ are difficult to obtain and even more difficult to standardize. Indeed, the healing of the skin is influenced by several parameters and factors that are dependent on the patient’s age, circulation, nutrition, or resistance to infection.^10^ In addition, for ethical reasons, it would not be possible to medically produce an artificial burn it in healthy subjects and thus standardize an accidental burn.

To overcome the limitations of in vitro and in vivo models, ex vivo models have been created. They have the advantage of being easily reproducible, less expensive and close to biological and clinical reality. Various methods have been used to create burns, each with not only advantages but also limitations. Scalds lack reproducibility, due to air bubbles creating non-uniform burns.^11^ Contact injury models [heated aluminum,^12–16^ heated brass or steel,^11,17–26^ electricity^27,28]^ are relatively affordable but require complex technical setups, thus making the size, shape and depth of the burns difficult to standardize. Some models have been created using lasers of various types: laser light (Thulium CW laser,^29^ CO_2_ laser,^30,31^ mid-infrared diode laser,^32^ blue beam laser,^33^ pulsed dye laser (PDL).^8,34,35^

Although being expensive, lasers are more frequently used as a medical tool in hospital settings. Laser burns have the advantage of reducing the variability observed in previous burn models, are more reliable in terms of reproducibility and usability, creating uniform, user-independent burns of known degrees. Furthermore, laser burns mimic actual burn wounds by inducing protein denaturation, collagen coagulation, vasoconstriction, platelet aggregation, and tissue necrosis, through heat transfer.^36^ However, no ideal standardized experimental model of burns induced by lasers on human skin exists till date.

Laser *(Light Amplification by Stimulated Emission of Radiation)* therapy is based on the principle that tissues absorb light. Skin tissues may have different interaction with the laser beam, such as refraction of light, absorption, diffusion, and transmission. The laser-skin interaction can generate the following effects: thermal, photochemical, electromechanical, and photoablative. We will focus on the thermal effect mechanism that interests us in this study. The different skin and laser settings (fluence, beam diameter, and pulse duration) will determine the amount of damage created. It is possible to observe different effects on the skin depending on the light energy absorbed by the tissue, which is then transformed into heat and causes damage proportional to the increase in its temperature. First of all, there is a denaturation of proteins at 60°C, then a coagulation at 80°C which results in an irreversible necrosis, without loss of tissue. Finally, a volatilization, ie, a loss of tissue substance, beyond 100°C. When a laser has a sufficiently short emission time so that the heat does not have time to diffuse (ie, a time shorter than that of thermal relaxation, which is the time necessary for 63% of the heat gained by the tissue to dissipate), there will be a thermal accumulation that will create a dilation of the local tissues which will then not be able to expand because of the adjacent tissues. This will lead to local explosive vaporization.^37^ The PDL that will be used in this study has a wavelength of 595 nm, itself produces a mechanical effect (photothermolysis).

The goal of this work was first to determine the parameters of a PDL inducing first, second, and third degree burns on humans, especially superficial second degree and deep second degree burns. For this purpose, the induced burns were described using a newly created classification. Our second objective was to prove, by the observation of the re-epithelization, that our model is as close as possible to an in vivo burn.

## MATERIALS AND METHODS

### Human Skin Sample

**Figure 1.**
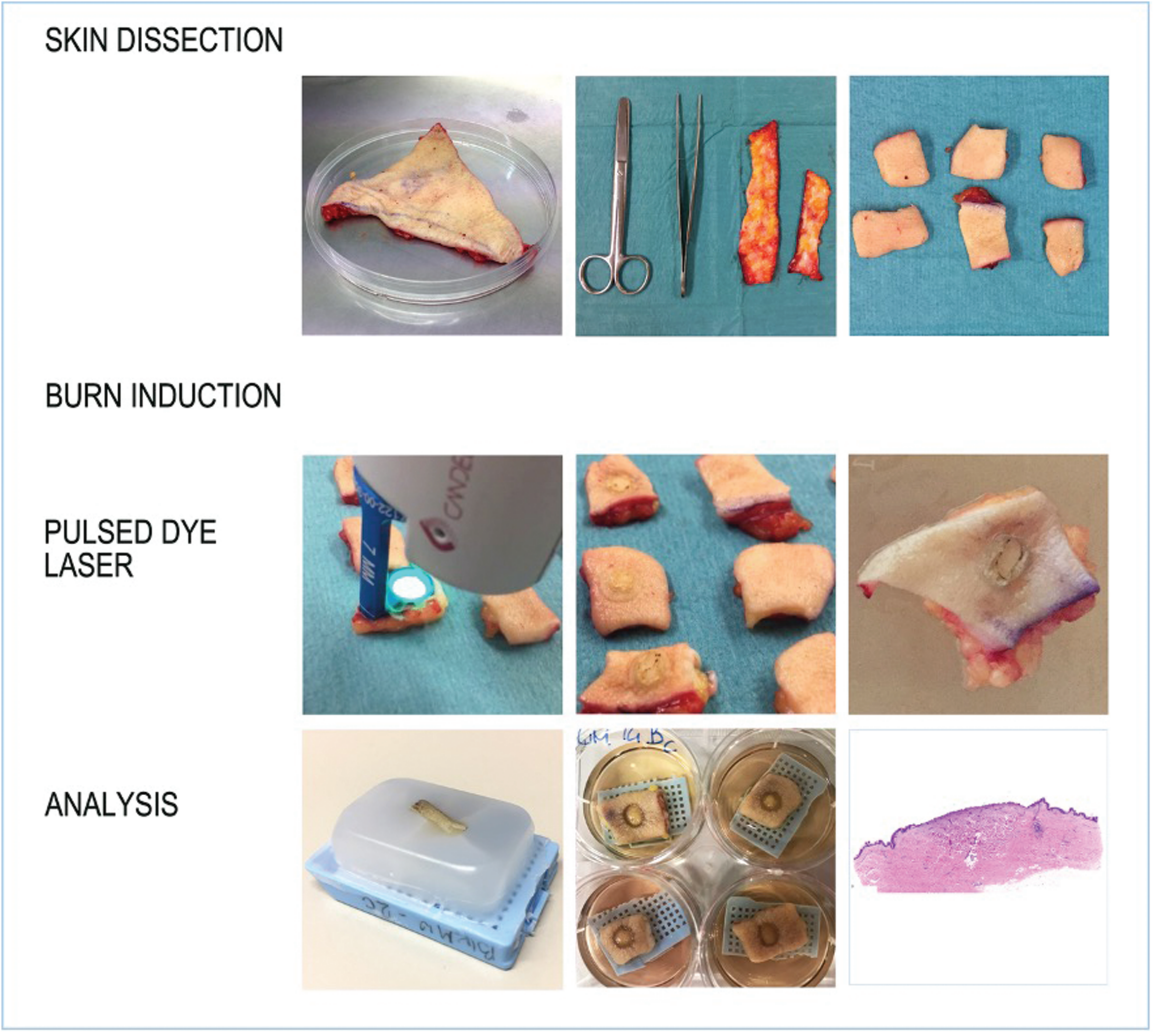
Experimental procedure. Fat was removed from excess abdominal skin samples and cut into 1 cm^2^ pieces. The burn was induced with a pulsed dye laser (PDL). After the burn was induced, skin samples were either fixed or cultured and finally histological and immune-histological analyses were performed.

Specimens of human abdominal full thickness human skin samples from six Caucasian females (age range 20–50 years, Fitzpatrick classification skin types II and III) were obtained as surgical waste from surgeries (Department of Musculoskeletal Medicine DAL) and as anonymous donation from the biobank, in accordance with its regulation and accepted by the Cantonal Ethics Committee under protocol 264/12. After surgery, skin samples were collected at the surgery desk and transported to the laboratory where it was prepared for the burn. The subcutaneous fat was removed as much as possible, leaving only a thin layer below the dermis (Figure 1). The thickness of the skin was 2.5 mm +/- 0.5 mm. The skin was then cut in 2 × 2 cm^2^ pieces with Mayo scissors and the burn wounds were induced on the epidermis of each sample, except for one sample which was used as a control. The burn lesions were induced on all skin samples within one hour after surgery. These parameters were kept fixed to avoid intra-experimental bias.

### Burn Device

The Syneron Candela V-beam PDL was used in this study. This laser has a wavelength of 595 nm, with a hand piece producing a 7 mm diameter beam. The fluence range of this laser can vary between 7 and 20 J/cm^2^, with a series consisting of 5–45 pulses. A pulse was delivered for a duration of 3–40 ms and was composed of micropulses generated at regular intervals of 100 μs. Laser safety glasses, masks and sterile gloves were used during the irradiation periods.

### Burn Wound Induction

For every donor, skin samples were cut in equally-sized 2 × 2 cm^2^ samples. Burns were then induced with the PDL by applying the hand piece directly on the epidermis of the 2 × 2 cm^2^ skin samples (see Figure 1). Two fluences were tested: 7 J/cm^2^, which corresponds to the clinical settings for the treatment of pediatric port wine stains,^36^ and 13 J/cm^2^ to test a higher fluence rate. We have varied the number of pulses from 5 to 45, and also their duration from 13 to 40 ms. For each fluence, we varied the number of pulses from 5 to 45 as well as their duration from 3 to 40 ms. For each parameter, a minimum of two skin samples from two donors were selected (up to five). Three replicates per donor were induced, for a total of six samples. Each experiment used skin tissue from two donors and was performed by two independent operators. This ensured the intra- and inter-donor reproducibility of results. Table 1 shows the different series performed according to the parameters of the chosen laser.

**Table 1.** **Pulse dye laser (PDL) laser parameters used for the experiments.** All experiments were performed with a wavelength of 595 nm and a handpiece producing a 7 mm diameter beam.

Pulse dye laser parameters			Number of donors n_D_	Number of experiments n_E_
Fluence (J/cm^2^)	Pulse duration (ms)	Number of pulses		
7	40	5	1	3
		10	2	6
		15	2	6
		20	2	6
13	3	5	2	6
		15	5	15
		30	5	15
13	40	30	2	6
		45	2	6

Samples were then either cut in the center of the phlycten and fixed for histology in 4% paraformaldehyde directly after the burn induction or transferred in Petri dishes to the laboratory for the air-liquid organo-culture.

### Histology and Immunohistochemistry

After 24 hours fixation in 4% paraformaldehyde, skin samples were rinsed once with phosphate buffered saline (PBS) and embedded in paraffin. Samples were oriented as shown in Figure 1. In brief, because the induced burns were circular in shape, samples were cut in half to place the center of the burn at the front. This also made it easier to assess burn depth. Cross sections of 5 μm were obtained with a microtome and allowed to dry for twenty-four hours on the slide. After de-paraffinization and hydration treatments, the slides were stained with Hematoxylin & Eosin (H&E) and Masson’s Trichrome. The skin components were stained with Eosin in different shades of pink, depending on their acidophilicity: the stratum corneum and keratinocytes of the epidermis appeared dark pink while the collagen of the dermis appeared pale pink and the cutaneous appendages and leukocytes in purple. Staining with Masson’s Trichrome allowed a precise delineation of collagen coagulation and thus of the burn itself. The collagen in the dermis was stained blue by this dye and turned maroon when it coagulated. The damage to the collagen, visualized with this dye, was a reliable method for determining the depth of a burn.^17^

Detection of proliferation (rabbit α-Ki67, SP6, Thermo Fisher MA5-14520, diluted 1:100) was performed using the fully automated Ventana Discovery ULTRA (Roche Diagnostics, Rotkreuz, Switzerland). All steps were performed with Ventana solutions. Briefly, dewaxed and rehydrated paraffin sections were pretreated with heat using standard condition (40 min) CC1 solution. The primary antibodies were incubated one hour at 37°C. After incubation with a rabbit Immpress HRP (Ready to use, Vector Laboratories, MP-7401), chromogenic revelation was performed with ChromoMap DAB kit (Roche Diagnostics, Rotkreuz, Switzerland). Sections were counterstained with Harris hematoxylin and permanently mounted. Ki67 immunohistochemistry was performed by the Histology Core Facility of the Swiss Federal Institute of Technology in Lausanne (EPFL), Switzerland.

### Burn Wound Depth Evaluation

**Figure 2.**
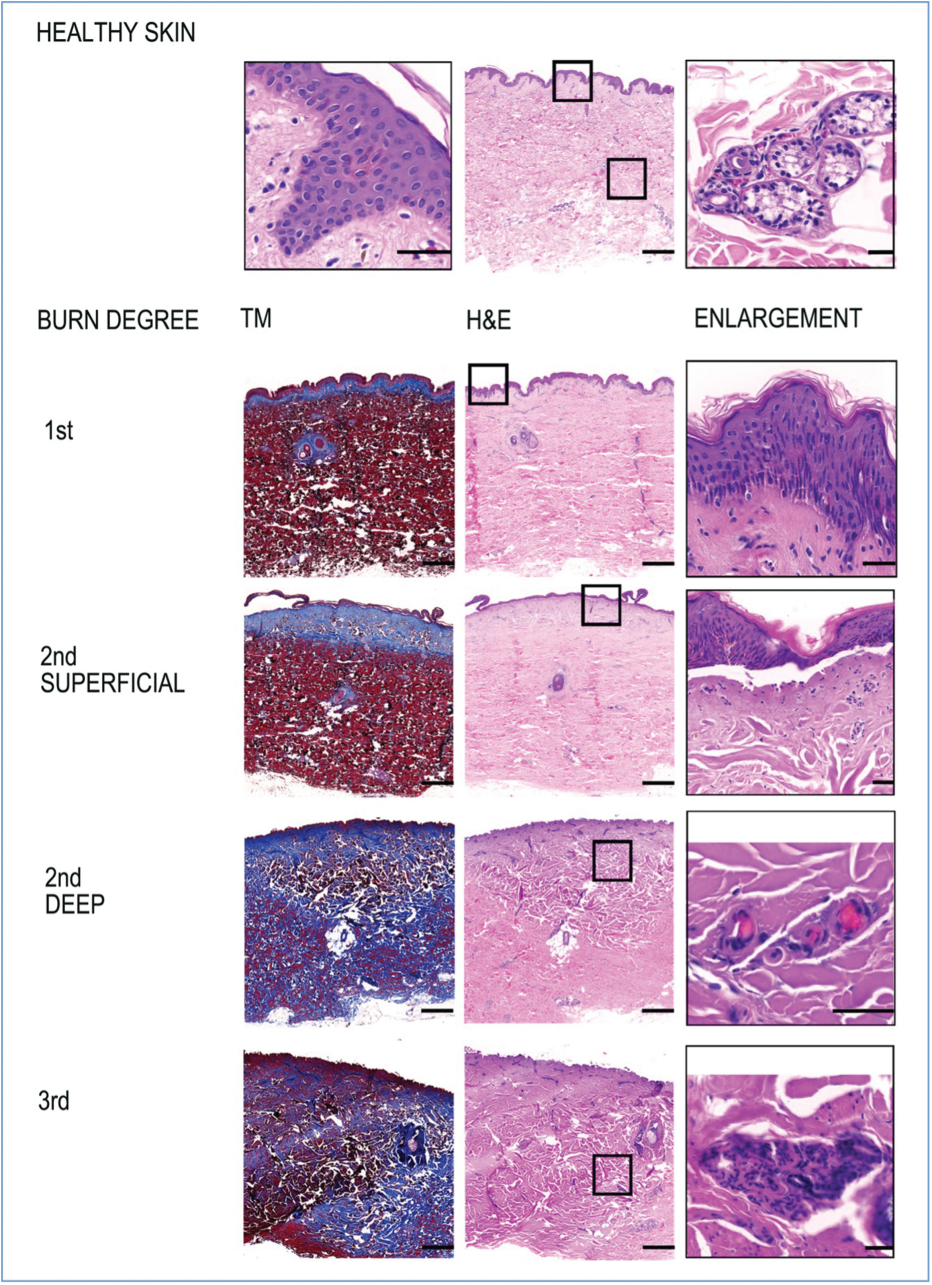
Illustration of burn depth evaluation with the classification code. Cross-sections of skin tissue stained using Trichrom of Masson and hematoxylin and eosin embedded in paraffin. First degree burn: Vacuolization & elongation of epidermal keratinocytes. Superficial second degree burn: Partial epidermal basal layer separation and coagulation of collagen in the papillary dermis. Deep second degree burn: Complete epidermal ablation, coagulation of collagen in the papillary and reticular dermis, thrombosis, partial destruction of skin appendages. Third degree burn: Necrosis of all layers down to the hypodermis and total destruction of skin appendages. Scale bar main image: 1000 µm, scale bar enlargement image: 40 µm.

The histological slides were first read by a dermatologist, a biologist and a medical doctor blinded to experimental irradiation parameters so that the assessment of the degree of the burn was not influenced by prior knowledge of the illumination conditions. In a second step, the histological assessments were pooled, and the hypotheses thoroughly discussed. Finally, with all the data taken together, a model of ex vivo evaluation of burn depth was determined. The analysis of burn depth was based on the loss of structure of the elements (keratinocytes, basal layer, glands, hair follicles, blood vessels) found in each layer of the skin, loss of substance, and also the coagulation of collagen (see Figure 2).

Slides were observed with a Leitz Laborlux 12 microscope and a Leitz DMRB microscope. Pictures were taken with a Leitz DMRB microscope, a Leica M205 FA Stereomicroscope and whole slide scans were done with a Zeiss Axioscan Z1 at the Cell Imaging Platform.

### Air–Liquid Skin Explant Culture

After the burn induction, skin samples were placed on top of sterilized histology cassettes in 6-well dishes (see Figure 1). Keratinocyte Green culture medium^37,38^ consisting of Dulbecco’s Modified Eagle Medium (DMEM) and Ham’s F-12 at a 3:1 ratio, 20 μg/mL gentamicin, 0.14 nM cholera toxin, 400 ng/mL hydrocortisone, 8.3 ng/mL EGF, 832.2 μM glutamine, 100 U/mL penicillin, 100 μg/mL streptomycin, 0.12 U/mL insulin, and 10% Fetal Bovine Serum was added into the dishes, to obtain an air–liquid interface. Petri dishes were then incubated for 14 and 21 days at 37°C with 5% CO_2_ with medium changes every two days. After 14 and 21 days, samples were fixed in 4% paraformaldehyde and processed as described above in section

### Statistical Analysis

Data in Tables 4 and 5 are presented using contingency tables or bar graphs of the contingency table. For paired measurements, a McNemar test was applied on the contingency table. For non-paired measurements, a classical Chi2 test was calculated on the contingency table to estimate the dependency of the variable in the contingency table. A *p* value ≤ .05 was considered as statistically significant.

**Table 4.** Contingency table

Pulse dye laser parameters Fluence (J/cm^2^) = 7 Pulse duration (ms) = 40	Degree of the induced burn (number of samples)	
Number of pulses	2^nd^ superficial	2^nd^ deep
10	6	0
20	3	3

Chi2(6) = 29.4; *p* < .001.

Highly significant.

Contingency table representing the significant differences of the second degree burns related to the number of pulses with fixed fluence and pulse duration. McNemar test (Chi2(6) = 29.4; *p* < .001).

**Table 5.** Contingency table

Pulse dye laser parameters Fluence (J/cm^2^) = 7 Pulse duration (ms) = 3	Degree of the induced burn (number of samples)	
Number of pulses	2^nd^ superficial	2^nd^ deep
15	12	3
30	0	13

Chi2(6) = 52.5; *p* < .001.

Highly significant.

Contingency table representing the significant differences of the second degree burns related to the number of pulses with fixed fluence and pulse duration. McNemar test (Chi2(6) = 52.5; *p* < .001).

## RESULTS

### Burn Wound Depths Classification Code

We have developed a classification code for burn wound depth following different established parameters, which is summarized in Table 2.

**Table 2.** Classification code for burn wound depth.

Burn type	Histological parameter
First degree burn (superficial burn)	Partial necrosis of the epidermis with Vacuolization and elongation of epidermal keratinocytes Edema of the epidermis No involvement of the dermis or subepidermal detachment
Second degree superficial burn (partial thickness)	Necrosis of the entire epidermis Partial lesion of the basal layer Subepidermal bulla Involvement of the papillary dermis coagulation of collagen in the papillary dermis
Second degree deep burn (deep partial thickness)	Involvement of the reticular dermis Complete destruction of the basement layer Partial destruction of the adnexa (the more superficial adnexa are affected but the deeper ones may be spared) with necrosis and anarchic structures of certain adnexa Fusion of collagen branches in the papillary and reticular dermis Thrombosis
Full thickness	Necrosis of all layers down to the hypodermis All appendages are burned

We observed a histological change in the epidermis after first degree burns: vacuolization forms in the cytoplasm of the necrotic keratinocytes. It can be seen as a white space in Figure 2. At this stage of the burn, the basal layer is not completely destroyed and the epidermis is still in contact with the dermis.

As soon as a phlyctene (detachment of the epidermis) appears macroscopically, a second degree burn is observed on histology assessment. This epidermal-dermal bulla is easily observed on histological sections as space separating the first two layers of the skin. During a second degree burn, whether superficial or deep, a vertical elongation of the keratinocytes of the epidermis can be observed, as well as a coagulation of the collagen of the dermis. This coagulation appears in histology as a lens-shaped area, lighter in color than the rest of the dermis.

Superficial second degree burns are characterized by coagulation of the collagen in the superficial layer of the dermis (papillary dermis). The skin appendages within the reticular dermis are not affected in this grade of burn.

Histologically, a deep second degree burn differs essentially from a superficial second degree burn by a coagulation of collagen reaching the layers of the deep dermis (reticular dermis). The collagen bundles are thickened and fused and take on an eosinophilic appearance, ie, a more intense pink color. In such cases, some of the skin appendages may be partially destroyed, leading to an anarchic structure.

Third degree burns involve all layers of the skin, down to the hypodermis.

### Burn Wound Depths Regarding Laser Parameters

Table 3 summarizes the burn depths obtained with the various laser parameters.

**Table 3.** Burn depths obtained with our experimental setting.

Pulse dye laser parameters			Degree of the induced burn (number of samples)			
Fluence (J/cm^2^)	Pulse duration (ms)	Number of pulses	1^st^	2^nd^ superficial	2^nd^ deep	3^rd^
7	40	5	**3**	-	-	-
13	3	5	**5**	1	-	-
7	40	10	-	**6**	-	-
7	40	15	-	**6**	-	-
13	3	15	-	**12**	**3**	-
7	40	20	-	**3**	**3**	-
13	3	30	-	-	**13**	**2**
13	40	30	-	**1**	**5**	-
13	40	45	-	-	**5**	**1**

**Figure 3.**
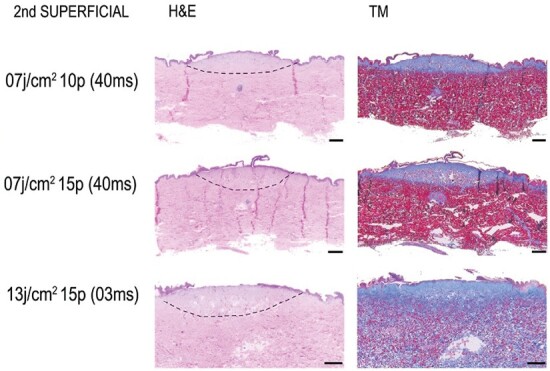
Induced second degree superficial burns with various pulse dye laser (PDL) parameters (fluence (J/cm^2^), pulse count (p) and pulse duration (ms). Dash lines represent burn areas. The scale bar represents 1000 µm.

**Figure 4.**
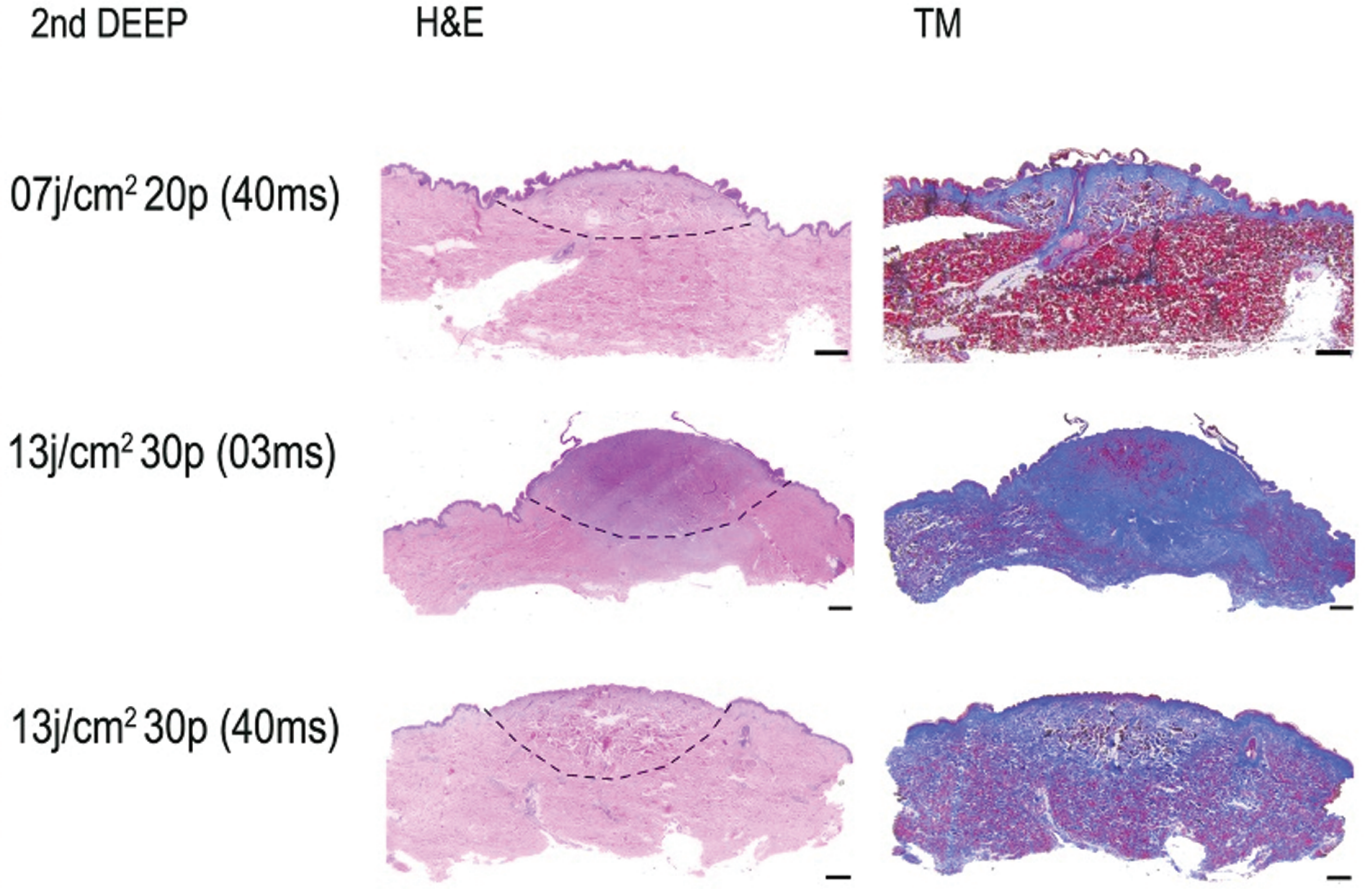
Induced second degree deep burns with different pulse dye laser (PDL) parameters (fluence (J/cm^2^), pulse count (p) and pulse duration (ms). Dash lines represent burn areas. The scale bar represents 1000 µm.

It is noteworthy that various combinations of parameters lead to the same burn depth. A first degree burn was obtained with 5 pulses, independently of the fluence rate or the pulse duration. Several parameters lead to the appearance of a second degree burn as shown in Table 3. We can clearly see that the fluence or the pulse duration has little effect on the creation of the burn. A second degree superficial burn was obtained with 10–15 pulses (see Figure 3) and a second degree deep burn needed 20–45 pulses (see Figure 4). The appearance of the burn was quite the same as observed with a H&E or MT coloration shown in Figures 3 and 4.

First and third degree burns are more extreme and are thus more easily induced with the PDL. However, second degree superficial and deep burns are not only difficult to create but also difficult to reproduce. We have therefore statistically analyzed the relationship between the increase in the number of pulses and the increase in the burn grade for these specific burn grades with two different fluences.

**Figure 5.**
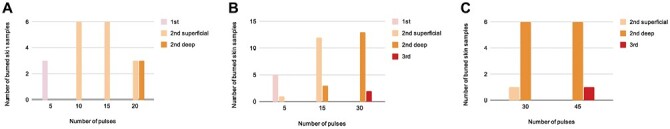
(A) Pulse dye lasers parameters: fluence: 7 J/cm^2^, pulse duration: 40 ms. Burn depth showed a statistically significant dependence relationship with increasing pulse counts. (B) Pulse dye lasers parameters: fluence: 13 J/cm^2^, pulse duration: 3 ms. Burn depth showed a statistically significant dependence relationship with increasing pulse counts. (C) Pulse dye lasers parameters: fluence: 13 J/cm^2^, pulse duration: 40 ms. Burn depth did not show a statistically significant dependence relationship with increasing pulse counts.

Burn depth showed a statistically significant dependence relationship with increasing pulse count (see Figure 5).

A superficial and deep second degree burn wound model was created with the same wavelength (595 nm), laser beam diameter (7 mm), fluence (7 J/cm^2^), and pulse duration (40 ms). A total of 10 pulses were necessary to induce a superficial second degree burn and 20 pulses for deep second degree burns. Demonstrated by a significant McNemar test (Chi2(6) = 29.4; *p* < .001) (see Table 4).

A superficial and deep second degree burn wound model was created with the same wavelength (595 nm), laser beam diameter (7 mm), fluence (13 J/cm^2^), and pulse duration (3 ms). A total of 15 pulses were necessary to induce a superficial second degree burn and 30 pulses for deep second degree burns. Demonstrated by a significant McNemar test (Chi2(6) = 52.5; *p* < .001) (see Table 5).

### Burn Wound Re-epithelization

Nouri et al. explained that long pulse times (longer than the relaxation time of the irradiated tissue) could induce more cellular damage around the burn because of heat diffusion.^39^ We can therefore suggest that our laser parameters, especially the pulse time, were too long in the first series with 40 ms of pulse duration. This induced higher cellular damage and did not allow a re-epithelialization of the injured area.

To create a burn wound model capable of being maintained in culture for several weeks, we therefore chose only deep and superficial second degree burns acquired with 3 ms pulse duration.

A superficial and deep second degree burn wound model was created with the same wavelength (595 nm), laser beam diameter (7 mm), fluence (13 J/cm^2^), and pulse duration (3 ms). A total of 15 pulses were necessary to induce a superficial second degree burn and 30 pulses for deep second degree burns.

**Figure 6.**
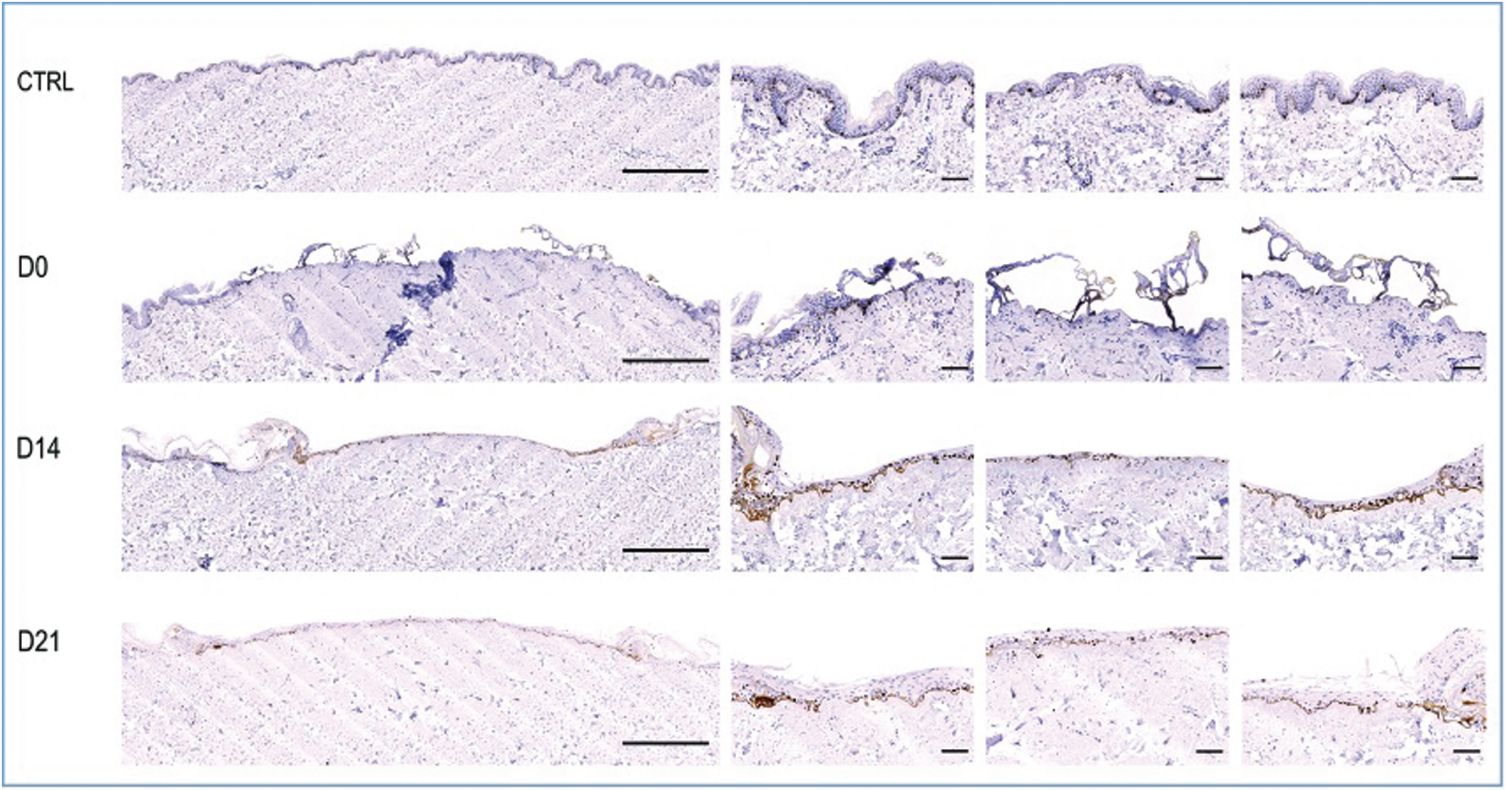
Cross-sections stained using Ki 67 antibody of skin tissue embedded in paraffin. Control (CTRL): healthy skin with keratinocyte proliferation. Day zero (D0): skin sample is fixed after inducing a burn, with complete epidermal ablation. Day fourteen (D14): after a 14-day culture, skin shows neo-epidermis and keratinocyte proliferation. Day twenty-one (D21): after a 21-day culture, skin samples show neo-epidermis and keratinocyte proliferation. Scale bar main image (left): 1000 µm, scale bar detail image (right): 100 µm.

To determine the viability of our deep second degree burn model, burned skin was maintained in an air-liquid interface for 14 and 21 days. At 14 days, we observed a re-epithelization visible with Ki67, with the creation of neo-epidermis. We have observed that the neo-epidermis thickness had increased at 21 days and that this phenomenon was more pronounced near the edges of the burned region. Moreover, the organized alignment of keratinocytes formed a stratum corneum (see Figure 6).

## DISCUSSION

Our study shows that PDL illumination is a reliable, rapid, reproducible, user-independent, and ethical method of inducing burns on ex vivo human skin samples. This work allowed us to determine, for the first time, the PDL illumination parameters inducing all grades of burns, including superficial and deep second degree. It also allowed us to create superficial and deep second degree burns with relatively short pulse durations of 3 ms, allowing a greater precision of the injured skin area. A total of 15 pulses were necessary to create a superficial second degree burn and 30 pulses for a deep second degree burn, with fixed parameters (wavelength: 595 nm; fluence: 13 J/cm^2^; laser beam diameter: 7 mm). In addition, our study demonstrated that these burn models on human skin can form a neo-epidermidis in an air–liquid interface culture.

We chose to apply the parameters of PDL that are used for the treatment, in children and in our hospital, of congenital angioma/port-wine stains (PWS) which are low-flow vascular malformations of the skin.^40,41^ Therefore, short pulse duration (3 ms) was employed, as we observed that with longer pulse parameters (40 ms) tissue and cellular damage around the burn area was higher. Moreover, this cellular damage did not allow re-epithelialization of the injured area (data not shown). This phenomenon of heat diffusion was also described by Nouri et al.^39^ It is for this reason that we maintain the laser parameters with a fluence of 13 J/cm^2^ and a pulse time of 40 ms. One important point to note is that the skin has to be burned at room temperature one hour after the surgery to avoid a change of burn degree when using similar parameters.

An ex vivo model has no blood circulation and therefore cannot generate real inflammatory responses. It should also be noted that our results may differ from in vivo laser experiments because the laser beam is not preferentially absorbed by the blood in the vessels in our model. In addition, there are fewer chromophores available in ex vivo skin and this whole process takes place at a temperature lower than normal body temperature. Cooler skin at room temperature will indeed undergo damage at a higher irradiation dose than an in vivo model.^34,42^ This study was conducted in the context of breast reconstruction, which implies only adult female subjects and the use of abdominal skin. These elements may influence the generalizability of the model but not our conclusions. The difficulty to cut circular wounds in the middle was circumvented by cutting through a circular erythema (for first degree burns) or a phlycten (for second degree burns), which systematically appeared on the skin upon laser irradiation.^43^

The central point in this study was to determine if the chosen system could induce second degree burns in a reproducible way. The same laser parameters were used on a minimum of six skin samples and two donors to assess intra-experimental reproducibility. The same laser parameters were also applied to different skin samples from different series to observe the reproducibility between experiments. We observed a user independent reproducibility in our experiments. A future study may confirm reproducibility through a larger panel of experiments and statistical analyses.

Till date, few studies have included ex vivo superficial and deep second degree burn models.^9,44^ This specific point is a major concern for the clinicians because these two degrees of burns require different management. Indeed, the healing potential of burns is directly related to the depth of the injury. Conservative management is possible for superficial second degree burns, whereas surgical management is necessary for deep second degree deep burns, as the burn has destroyed the cells capable of regenerating the superficial layers of the skin. The use of our model would be of interest to evaluate the efficacy of different cellular treatments to treat burns such as artificial skin, cultured keratinocyte sheets/dermo-epidermal skin grafts or allogenic covers.

We were able to show that our burn model remains viable in an air–liquid interface for at least 21 days. Moreover, keratinocytes adjacent to the burn can divide and migrate to reform an epidermis mimicking an in vivo burn. In a future study, it would be interesting to perform a quantitative analysis of keratinocyte proliferation over several days.

## CONCLUSION

In conclusion, the majority of current skin burn wound models are animal models (especially the porcine model). Although porcine skin is similar to human skin in many respects, this model has both anatomical and physiological limitations. In vitro models represent an interesting new alternative, but do not sufficiently reflect the complexity of in vivo skin. Some studies use ex vivo human skin samples but very few studies attempted to create a model of superficial and deep second degree burns. Furthermore, none of these studies used a laser to standardize the burn wound induction. Our results show that the novel ex vivo model used on human skin with a PDL is specific and reproducible of second degree superficial and deep burns, which resemble clinical reality. This model also has the advantage of being easily and quickly reproducible and user-independent. In addition, it can be used to evaluate the pathophysiology of burns. Moreover, it can foster the transition from animal to nonanimal experiments. Overall, we are convinced that this model can serve as a basis to develop new treatments for burns and their subsequent management to improve patient care.
